# Pioneering evaluation in Brazil of microscope-integrated optical coherence tomography with a three-dimensional digital visualization system during pars plana vitrectomy for the treatment of macular hole

**DOI:** 10.1186/s40942-025-00671-8

**Published:** 2025-05-19

**Authors:** Leticia Pinheiro de Freitas, Jamil Miguel Neto, Laís Lauria Neves, Thais Bastos, Alexandre Caiado Ferreira Pires, Antônio Marcelo Barbante Casella, David Leonardo Cruvinel Isaac, Marcos Pereira de Ávila

**Affiliations:** 1 Ophthalmology Reference Center at the Federal University of Goiás, Goiânia, Go 74605-020 Brazil; 2 Brazilian Eye Surgery Center, Goiânia, GO 74210-010 Brazil; 3 Department of Ophthalmology, State University of Londrina, Londrina, PR 86057-970 Brazil

**Keywords:** Macular hole, Vitrectomy, iOCT, ILM peeling

## Abstract

**Background:**

This study aimed to characterize the clinical results and usability of intraoperative optical coherence tomography (iOCT) associated with a digital visualization system in vitreoretinal surgery for macular hole correction.

**Methods:**

This is a descriptive observational study of patients undergoing vitreoretinal surgery for macular hole at Brazilian Eye Surgery Center in which the digital visualization system associated with iOCT was used. Anatomical and functional results were collected 6 months after surgery. Macular hole closure rates, pre- and post-operative visual acuity were measured in addition to surgeon feedback and the percentage in which the technology allowed intraoperative decision-making.

**Results:**

25 eyes of 25 patients were included in the study. The mean preoperative visual acuity was 20/100, ranging from 20/50 to less than 20/400, and postoperative visual acuity was 20/60, ranging from 20/25 to less than 20/400. The time spent with iOCT did not result in surgical delay, as the average time spent was 3.24 extra minutes spent per surgery. Closure of the macular hole was achieved successfully in 92% of cases. In 8% of them surgical success in closing the macular hole was achieved after a new fluid-gas exchange in the office. The surgeon preferred real-time iOCT and, in 4% (1/25) of cases, it influenced the change in surgical technique, preventing unnecessary ILM (Internal Limiting Membrane) peeling after complete removal of the posterior hyaloid and closing the macular hole. In all cases the surgeon reported valid feedback regarding the use of information provided by real-time OCT. This tool was valid for confirming complete ILM peeling in all cases in which it was performed. Finally, iOCT made it possible to identify the appropriate location to begin creating the ILM flap in 2 cases (8%) both of fragile retina; It prevented a new injection of dyes to identify residual ILM in 8% of cases (2/25) and allowed verification of the correct positioning of the pedicled ILM flap over the hole in 16% (4/25) of cases. Therefore, in 36% of cases (9/25) iOCT was essential for the final surgical outcome.

**Conclusion:**

This study suggests that the use of iOCT integrated with a digital viewing microscope for the treatment of macular holes offers high standard usability and effectiveness for visualizing structures, and impact favorably on decision-making process during pars-plana vitrectomy. These findings suggest that in the near future, expanded use of iOCT could significantly improve tissue management at the vitreomacular interface and improve anatomical and functional results.

**Supplementary Information:**

The online version contains supplementary material available at 10.1186/s40942-025-00671-8.

## Background

Advances in instruments and visualization systems for the retina and vitreous have made modern vitreoretinal surgery possible. In 1946, Perritt first used a bench-mounted binocular microscope in ophthalmic surgery [1]. In 1952, Littmann of Carl Zeiss, Inc. developed a binocular microscope equipped with coaxial illumination for use as a colposcope and otoscope. It became commercially available in 1953 and was immediately adapted for eye surgery by Harms in Germany. Later that same year, it was used by Barraquer in Buenos Aires during the 10th Argentine Ophthalmological Congress. Such advancements allowed for more detailed visualization of ocular structures [2].

The introduction of these technologies, along with changes in fluid systems with controlled aspiration, increased cutting speeds of vitrectomy cutters, and especially small-gauge instrumentation, brought further improvements, safety, and effectiveness to vitreoretinal surgical techniques [3].

Over the past four decades, advances in computing systems have transformed the way images are captured and used in medicine, transitioning to digital formats. Ophthalmology has been one of the specialties that was most benefited from this progress, which underpins most complementary diagnostic exams in its subspecialties. Within this new technological arsenal, Optical Coherence Tomography (OCT) emerged. This paradigm shift highlighted the need to rethink how these instruments are used, how new techniques should be developed, and their implications for novel surgical approaches [4].

Optical Coherence Tomography (OCT) plays an essential role in the investigation, diagnosis, and management of vitreoretinal pathologies. OCT enables the individualization of retinal layers and the detailed characterization of the vitreoretinal interface with high levels of quality. Furthermore, multimodal evaluation has proven fundamental in assessing and interpreting findings of the neurosensory retina and retinal pigment epithelium (RPE). Due to its significant importance in clinical practice, the need to bring these same advances and image quality into surgical practice has become indispensable [5].

The introduction of the 3D digital microscope coupled with iOCT in Brazil raised the necessity of testing this new technology, already recently employed in other countries, within our local context [6].

No studies have been published in the Brazilian literature evaluating the combined use of digital visualization technology and intraoperative OCT (iOCT) during pars plana vitrectomy for the treatment of macular hole until now. This study was designed to assess the application, safety, effectiveness, and usability of iOCT in this condition, evaluating its visual resources, software, time efficiency, and intuitive and safe use by surgeons.

This study aims to test and evaluate the use of intraoperative optical coherence tomography combined with a digital visualization system in macular hole surgery, focusing on its application, safety, usability, learning curve, and impact on changes to intraoperative surgical techniques.

## Materials and methods

### Research project

This is a descriptive observational study of patients undergoing vitreoretinal surgery for macular hole at the Brazilian Eye Surgery Center (CBCO) by a single surgeon, one of the authors of this study (MPA), in Goiânia, Goiás, Brazil. There was no sample size calculation as it was a case series.

This study adhered to the tenets of the Declaration of Helsinki and was approved by the institutional review boards of the participating institutions. All subjects voluntarily agreed to participate by signing the informed consent form.

The sample consisted of 25 eyes of 25 patients with macular holes who underwent vitreoretinal surgery combined with internal limiting membrane peeling and other maneuvers at the vitreomacular interface, performed using an exclusively digital system combined with intraoperative OCT during the period from March 2022 to August 2023.

### Inclusion criteria

The study was conducted with male and female patients, olden than 18-years old, with macular hole of any etiology.

### Exclusion criteria

Patients under 18 years of age, those who lost postoperative follow-up, patients who underwent surgery performed by a different surgeon, or surgeries in which iOCT was not used, were excluded.

### Ophthalmic evaluation

Patients underwent a complete ophthalmologic examination and anamnesis, including visual acuity measurement, anterior and posterior segment bio microscopy, fundoscopy, intraocular pressure measurement, and pre- and postoperative imaging (retinography, optical coherence tomography, and fluorescein angiography, when necessary). Initially, 35 patients were selected, with 25 patients completing the study. Due to loss of follow-up, 10 patients were excluded from the analysis.

Visual acuity determination was carried out using the Snellen optometric scale, which was then converted into a logarithmic scale for statistical analysis. Intraocular pressure was measured with a Goldman applanation tonometer after instillation of a single drop of 5 mg/mL proxymetacaine anesthetic eye drop. Visual acuity and intraocular pressure measurements were taken at all follow-up visits.

Fundoscopy was performed after pharmacological mydriasis with instillation of 1% tropicamide and 2.5% phenylephrine eye drops. One drop of each was administered with a five-minute interval in each eye, 40 min before the exam. Retina mapping was done using a binocular indirect ophthalmoscope (OBI) model OSF 1.0 (Eyetec^®^) with a super LED light source and a 20 diopter (Volk) double aspheric condensing lens, allowing for a complete exam with proper peripheral evaluation.

Wide-angle color fundus photography [7] was performed to provide photographic records of the retinal condition for medical documentation and to assess patient evolution. Spectral domain optical coherence tomography (SPECTRALIS^®^, Heidelberg Engineering), Germany was also conducted, a non-invasive, contactless imaging modality that produces micrometric resolution images of tissues.

Macular hole classification was based on the CLOSE Study criteria [8], with measurement of the macular hole and the presence/absence of vitreomacular traction or epiretinal membranes, as proposed by the IVTS (International Vitreomacular Traction Study) classification. The macular holes’ sizes were classified as follows:

1. Small macular hole (≤ 250 µm);
2. Medium macular hole (> 250 µm and ≤ 400 µm);
3. Large macular hole (> 400 µm and < 550 µm);
4. Very large macular hole (≥ 550 µm and < 800 µm);
5. Extra-large macular hole (≥ 800 µm and < 1000 µm);
6. Giant macular hole (≥ 1000 µm).

A descriptive analysis of the cases was performed, all carried out by the same surgeon (MPA, one of the authors of this work) using the same technique. The surgery was performed under pupil dilation, local anesthesia, and sedation at the CBCO surgical center, where the Constellation^®^ vitrector (Alcon) and the Carl Zeiss^®^ Artevo 800 microscope, with integrated intraoperative OCT (Carl Zeiss Meditec, Jena, Germany), were available and used. Depending on the surgeon’s case-by-case evaluation, expandable gas (C3F8) or silicone oil was used as vitreous substitutes.

All patients underwent 25-gauge pars plana vitrectomy. After core vitrectomy and posterior hyaloid detachment (if there was no preoperative posterior hyaloid detachment), the Opht-Blue^®^ dye (Ophtalmos) was used to stain the internal limiting membrane (ILM). ILM peeling was performed circumferentially around the macular hole using the Finesse Sharkskin ILM Forceps^®^ (Alcon), employing the “pinch and peel” technique, as preferred by the surgeon, in all cases where peeling was performed. A fluid-air exchange was carried out, and tamponade was performed with silicone oil or perfluoro propane gas, according to the surgeon’s decision, with the patient maintaining a face-down position for seven days postoperatively. In cases where macular hole closure was not achieved, a fluid-gas exchange was performed in the office after the instillation of anesthetic eye drops and the placement of an eyelid speculum. A scleral puncture was made in the pars plana using an insulin needle connected to a 10 ml syringe containing a 15% C3F8 gas mixture. Small amounts of liquid vitreous were slowly aspirated, collecting at the bottom of the syringe, followed by the injection of an equal amount of the gas mixture into the vitreous cavity.

In cases of recurrent or persistent macular holes, referred by another surgeon after prior vitrectomy, the inverted flap technique was performed, as first described by our group in Brazil in 2020 [9, 10]. In cases of refractory and persistent macular holes, where a large portion of the ILM had been removed, ILM was removed from the posterior pole or the midperipheral retina, defined as an intraoperative free flap. The free flap was obtained and delicately positioned, anchored to the anterior surface of the macular hole after autologous blood [11] was placed on its surface. This blood was obtained from the venous retinal capillary circulation through a delicate maneuver with the Finesse Sharkskin ILM Forceps® (Alcon), under the surgeon’s control. After positioning the free flap, the flap was mixed with the autologous blood clot. Intraoperative OCT (iOCT) was used to observe the flap in the desired position. Subsequently, fluid-air exchange and tamponade with C3F8 gas or silicone oil were performed according to the surgeon's decision. In cases where the surgeon had difficulty visualizing the flap’s positioning, perfluorocarbon was used with a bubble delicately placed temporarily to maintain the flap in the correct position during the fluid-gas exchange.

iOCT was used at specific moments during the surgical procedure (before, during, and after the peeling). All images were obtained with the ARTEVO 800^®^ [12]. The iOCT does not have the spatial resolution to identify the ILM adherent to the Retinal Nerve Fiber Layer (RNFL), but once its peeling is initiated, it can be identified using iOCT. The iOCT has a spectral-domain technology with a wavelength of 840 nm and an acquisition speed of 27,000 A-scans per second. The acquisition parameters include an A-scan depth of 2.9 mm and 5.8 mm in tissue, an axial resolution of 5.5 μm in tissue, an adjustable scan length between 3 and 16 mm, and an adjustable scan rotation of 360° [12].

The information provided by iOCT was available in real-time or statically (during pauses in maneuvers). After surgery, an evaluative questionnaire (Annex I) was completed by the surgeon, assistant, and fellows regarding visualization, image quality, usability, impact on intraoperative decisions, ergonomic improvement, and teaching, as well as the need for multiple dye applications and observed postoperative complications. Between two and four fellows from the UFG and CBCO services were present in the operating room. They also completed the questionnaire (Annex I) to evaluate their learning experience, visualization index, and overall experience.

Patients were evaluated on the first postoperative day with anterior segment biomicroscopy and tonometry. They were re-evaluated on the seventh postoperative day with anterior segment biomicroscopy, tonometry, and fundoscopy. At the first and sixth months postoperatively, in addition to the previous cited, retinography and optical coherence tomography were also performed.

The positioning of the device and the assistant in the operating room, the importance of the assistant's position and expertise in the field, the surgeon's opinion on usability, maneuver visualization, and the difficulty of having the surgical field on the same screen as iOCT visualization were also observed.

### Statistical analysis

The data collected were stored in a database management system. The characterization of the patient profile was conducted using the frequency of categorical variables with absolute and relative frequency values, and descriptive statistics for numerical variables, with mean and standard deviation values. The normality of the data was assessed using the Shapiro–Wilk test. The comparison of visual acuity preoperatively and six months postoperatively was made using paired t-tests and McNemar’s test. The data were analyzed using the Statistical Package for Social Sciences (SPSS) software, version 26.0 (IBM Corporation, Armonk, NY, USA). The significance level was set at 5% (p < 0.05).

## Results

A total of 25 eyes from 25 patients were included in the study. Regarding gender distribution, 14 patients (56%) were male, and 11 (44%) were female. In terms of age, the mean age at diagnosis was 63.48 ± 9.25 years, ranging from 40 to 80 years. Most patients were in the 60–69 age group (44%) (Table 1). Additionally, 92% patients underwent only one surgery for macular hole correction, while 8% (2/25) required additional gas fluid exchange in the office later. This procedure allowed complete gas-fluid exchange in the vitreous cavity.

**Table 1:** Characterization of Demographic Profile (n = 25)

Demographic profile	Descriptive statistics
Idade	
Years (Mean ± SD)	63.48 ± 9.25
Age range n (%)	
40–59 years	7 (28.0)
60–69 years	11 (44.0)
70 or more	7 (28.0)
Sex n (%)	
Male	14 (56.0)
Female	11 (44.0)

*n* Absolute frequency, *%* relative frequency, *DP* standard deviation

Source: Research Data

The mean preoperative visual acuity was 20/100, ranging from 20/50 to worse than 20/400, and the mean postoperative visual acuity was 20/60, ranging from 20/25 to worse than 20/400 (Table 2).

**Table 2:** Characterization of the clinical profile (n = 25)

Clinical profile	Descriptive statistics
Vertical diameter (Mean ± SD)	455.78 ± 140.28
Base (Mean ± SD)	783.19 ± 298.69
Smallest diameter (Mean ± SD)	411.18 ± 153.16
Closed MH n (%)	
No	2 (8.0)
Yes	23 (92.0)
Ocular comorbidities n (%)	
No	10 (40.0)
Yes	15 (60.0)
Which ocular comorbidities n (%)	
ERM	7 (43.8)
Others	9 (56.3)
Trauma n (%)	
No	24 (96.0)
Yes	1 (4.0)
Crystalline lens status n (%)	
Phakic	14 (56.0)
Pseudophakic	11 (44.0)
Macular hole classification n (%)	
Stage 1	5 (20.0)
Stage 2	9 (36.0)
Stage 3	5 (20.0)
Stage 4	6 (24.0)
Stage 5	0
Stage 6	0
Vitreous status: tractional component present n (%)	
No	20 (80.0)
Yes	5 (20.0)
Tamponade agent n (%)	
C3F8	23 (92.0)
Oil	2 (8.0)
Complications n (%)	
No	21 (84.0)
Yes	4 (16.0)

*n* Absolute Frequency, *%* Relative Frequency, *SD* standard deviation

Source: Research Data

Macular hole classification was based on the CLOSE study, and the number of eyes in each category was:

1. Small macular hole: 5 eyes
2. Medium macular hole: 9 eyes
3. Large macular hole: 5 eyes
4. Very large macular hole: 6 eyes
5. Extra-large macular hole: 0 eyes
6. Giant macular hole: 0 eyes

Ocular comorbidities found included cataracts, epiretinal membranes, retinal detachment, age-related macular degeneration, and high myopia.

**Fig. 1:**
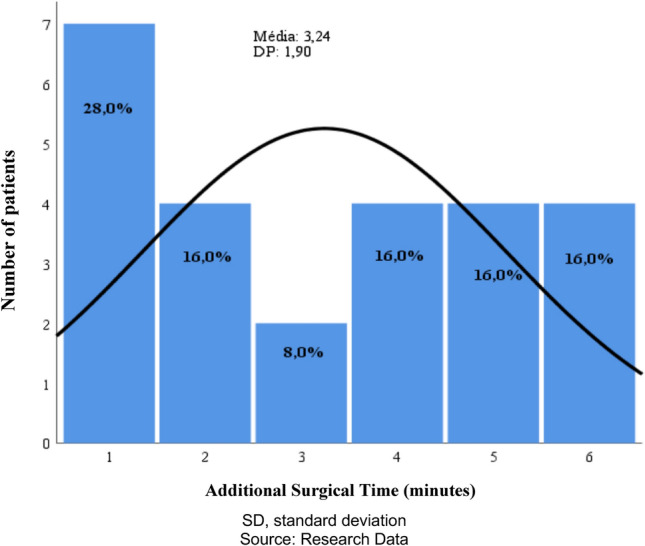
Histogram showing the descriptive statistics and distribution of the additional surgical time spent for performing iOCT

The average additional time spent by using iOCT was 3.24 min per surgery, with a standard deviation of 1.90 min (Fig. 1). Over time, as the technology was used, a reduction in time was noted, with the extra time reduced to an average of one additional minute by the end of the surgery. No intraoperative complications were associated with the use of this technology, and there were no adverse events or technological failures reported during the surgeries. No issues were encountered with the microscope, including technological failure, or challenges in obtaining images.

**Fig. 2:**
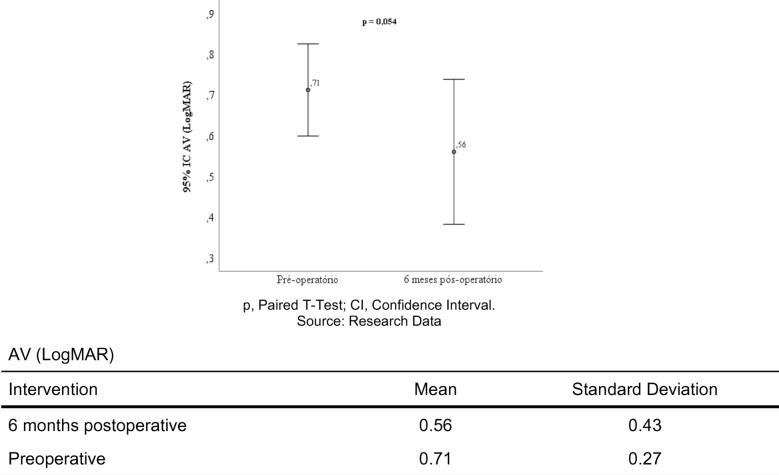
Results of the comparison of visual acuity pre-operatively and six months post operatively

Table 3 and Fig. 2 shows that in the preoperative period, there were no patients with visual acuity better than or equal to 20/25, with 16% of patients finishing the study with 20/25 visual acuity (p = 0.022). The same can be said for patients who finished the study with visual acuity of 20/40, with 28% of patients having this visual acuity after six months of surgery (p = 0.043). Furthermore, 16% of patients started the study with visual acuity of 20/100 and no patient finished the study with this visual acuity (p = 0.031).

**Table 3:** Results of the comparison of visual acuity in the preoperative and 6-month postoperative periods. (n = 25)

	Intervention		p*
	Preoperative n (%)	6 months postoperative n (%)	
AV (Snellen)			
20/25	0 (0.0)	4 (16.0)	**0.022**
20/30	1 (4.0)	1 (4.0)	0.612
20/40	0 (0.0)	7 (28.0)	**0.043**
20/50	2 (8.0)	2 (8.0)	0.989
20/60	4 (16.0)	1 (4.0)	0.121
20/80	6 (24.0)	3 (12.0)	0.102
20/100	4 (16.0)	0 (0.0)	**0.031**
20/150	2 (8.0)	0 (0.0)	0.871
20/200	4 (16.0)	4 (16.0)	0.982
20/400	2 (8.0)	2 (8.0)	0.899
20/800	0 (0.0)	1 (4.0)	0.812

^*^McNemar Test; *VA* visual acuity, *n* absolute frequency, *%* relative frequency

Source: Research Data

In each case, iOCT was successfully performed without incidents. The primary surgeon reported that the presence of a specialist assistant surgeon was crucial for continuous observation of iOCT and reviewing critical points during surgeries. In 4% (1/25) of the cases, iOCT prevented unnecessary ILM peeling after complete removal of the posterior hyaloid and resulted in macular hole closure during surgery and follow-up visits. In all cases, the surgeon provided valuable feedback regarding the use of real-time OCT information. This tool helped in confirming complete peeling in all cases where the ILM was peeled. IOCT enabled the identification of the appropriate location for initiating the ILM flap in 8% (2/25) of cases, particularly in fragile retinas; it also eliminated the need for additional dye injections to identify residual ILM in 8% of cases (2/25), and allowed verification of proper ILM flap positioning over the macular hole in cases of inverted or free flap techniques in 16% (4/25) of cases. Thus, in 36% of the cases (9/25), iOCT was considered highly important for the final surgical outcome.

The questionnaire completed by the fellows observing the surgery received unanimous responses, demonstrating a high rate of observation and utilization. The questionnaires completed by the primary surgeon, assistant, and fellows confirmed the excellent usability and effectiveness of the technology. Additionally, in 36% (9/25) of cases, the presence of the assistant surgeon was very important for identification in real time of critical points and alerting the primary surgeon. All respondents rated the visualization, image quality, ergonomics, and improvement in teaching as a score 5.

## Discussion

Vitrectomy surgery is undergoing a significant technological revolution with the advent of 3D visualization systems. According to the surgeons' perception, there has been an improvement in ergonomics, easier teaching, greater depth and resolution of images, and an expanded field of view. However, the integration of images and data, such as in the use of iOCT—Intraoperative Optical Coherence Tomography—requires adjustments and changes to improve image quality through controls by the surgeon and circulating staff in the operating room, resulting in additional time for the surgical procedure. In the context of introducing iOCT, the surgeon must train to acquire their own images, instead of receiving them ready for interpretation at the office. This set of factors leads to an increase in surgical time. In this study, the use of iOCT integrated with digital image visualization resulted in increased surgical time, with an average of 3.24 additional minutes per surgery for the use of intraoperative OCT technology, that did not cause surgical delays. This additional time was associated with the time required to turn on and initiate the OCT scans, adjust depth (Z-axis), conduct detailed studies with movement of the scanning beams to evaluate specific areas, and turn off the OCT to move the image in the surgical field. Over time and with the use of technology, an optimization of the iOCT image acquisition process was noted, with a reduction in the time spent. The average additional surgical time became approximately one to two minutes, about half the initial average time of 3.24 min. This reduction in time was attributed to the increased familiarity of the team with the technology.

The average total surgical time for the surgeon (MPA) to perform posterior vitrectomies associated with ILM peeling without using iOCT was 38.7 min, and with iOCT, it was 42 min. Thus, the additional surgical time for using the technology represents 7.7% of the total surgical time.

Recently, there have been significant advancements in pars plana vitrectomy technology, ranging from smaller gauge probes to fully integrated systems with the surgical microscope. Intraoperative optical coherence tomography (iOCT) has proven to be useful in diseases affecting the vitreoretinal interface, such as epiretinal membranes and macular holes, as well as in assessing the subretinal space during vitrectomy. It provides real-time, high-resolution cross-sectional imaging of the retina during surgery, allowing surgeons to visualize retinal structures and monitor tissue changes dynamically. Therefore, surgeons can check for residual membranes or tractions, allowing surgical decision-making and reduced complications [13].

Since its development, retinal OCT has become one of the most important tools for diagnosing and monitoring retinal diseases and has been used for both clinical and research purposes [14]. The present study adds information about the utility of a digital microscope integrated with an iOCT system for the treatment of macular holes, with an analysis of clinical outcomes up to six months post-surgery.

The technology demonstrated excellent usability and was essential in making important intraoperative decisions. Furthermore, no intraoperative complications were associated with the technology, showing not only its efficacy and efficiency but also a high level of safety. With the optimization of OCT integration in the intraoperative setup and the additional time at the end of surgery being considered insignificant by the surgeon (MPA), intraoperative feedback further prevented unnecessary surgical maneuvers, thereby reducing the risk of iatrogenic injuries.

Intraoperative OCT (iOCT) is an emerging OCT modality that allows real-time OCT imaging of the retina to be displayed for the surgeon during surgery. There are important limitations to be considered: the learning curve of the surgeons that need to adapt to integrating iOCT into their workflow, the high cost of this technology and the surgical instruments or fluid interfaces may introduce artifacts, affecting image quality.

The quality of ILM visualization may vary depending on the angle of the OCT beam relative to the retina and iOCT can detect residual ILM or incomplete peeled areas, which may not be visible with the microscope alone, enhancing surgical outcomes [13].

In the cases presented, situations were observed that greatly enhanced the use of the technology when the procedure was monitored in real time by the assistant surgeon, who must necessarily be an experienced surgeon and preferably able to better track vitreoretinal changes, exposing them to the primary surgeon during the procedure. When significant changes were found during the procedure, the assistant surgeon would report to the primary surgeon, who could use two mechanisms to review these changes. The first was to repeat the same maneuvers with the instruments to verify points of interest, such as vitreous traction, residual epiretinal membranes, or, less frequently, retinal ruptures. The second option was to review the images of the recorded points of interest for decision-making. In this way, iOCT assisted in making intraoperative decisions through direct observation, mainly by the assistant surgeon.

Modern macular hole surgery today is predominantly performed with ILM peeling, a procedure that has significantly increased macular hole closure rates [15, 16]. Currently, the inverted flap technique, described in Brazil by our group in 2020, is increasingly used for the closure of recurrent or persistent macular holes [9, 10]. In many cases, surgeons encounter difficulties in accurately positioning the flap. In some situations, it may even be necessary to use perfluorocarbon with a delicately and temporarily placed bubble to maintain the flap in the correct position during the fluid-gas exchange [17].

The observation of the inverted flap over the macular hole is typically achieved by staining the ILM with vital dyes, such as brilliant blue, and less commonly, indocyanine green. However, this observation can be challenging due to the low staining intensity of the ILM with these dyes in some cases, a difficulty that becomes more pronounced after the fluid-gas exchange.

The use of iOCT clearly demonstrates flap positioning during this surgical step, even in the presence of air in the vitreous cavity. This phenomenon is likely due to the persistence of a thin layer of balanced salt solution (BSS) on the vitreoretinal interface. This finding is reported here for the first time.

In 2020, our group reported that maintaining a non-prone head position postoperatively is also safe and yields good outcomes [18]. However, in the present study, a "face-down" head positioning was chosen for seven days post-surgery due to the multivariate etiologies of macular hole cases.

In this study, there were several emblematic cases that illustrate the usability of digital technology with iOCT incorporated into the microscope during vitrectomy surgery for macular hole treatment, where its use was essential for the final surgical success.

**Fig. 3:**
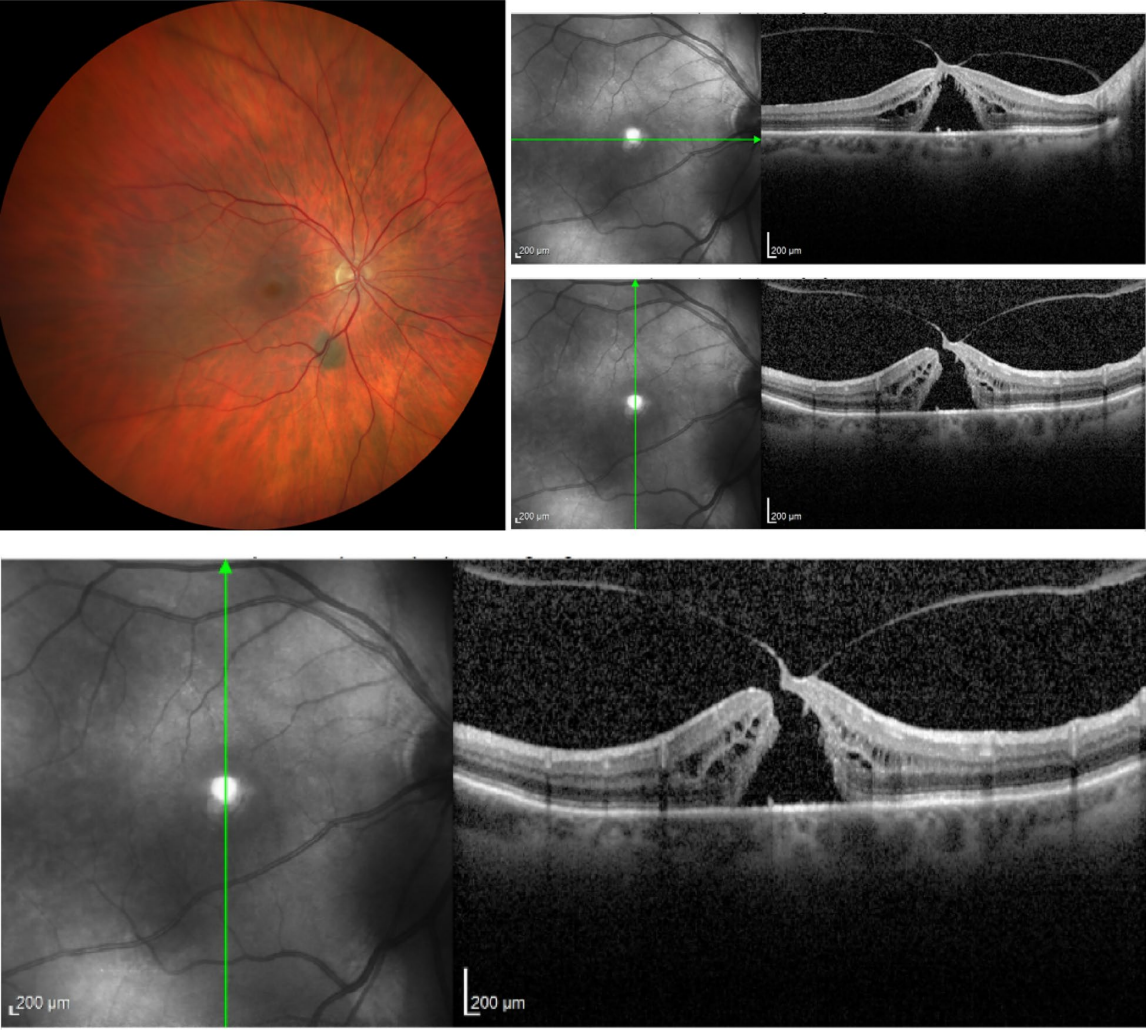
Case 1. Macular hole with vitreomacular traction. Retinography on the left and OCT images on the right and below in the pre-operative phase

**Fig. 4:**
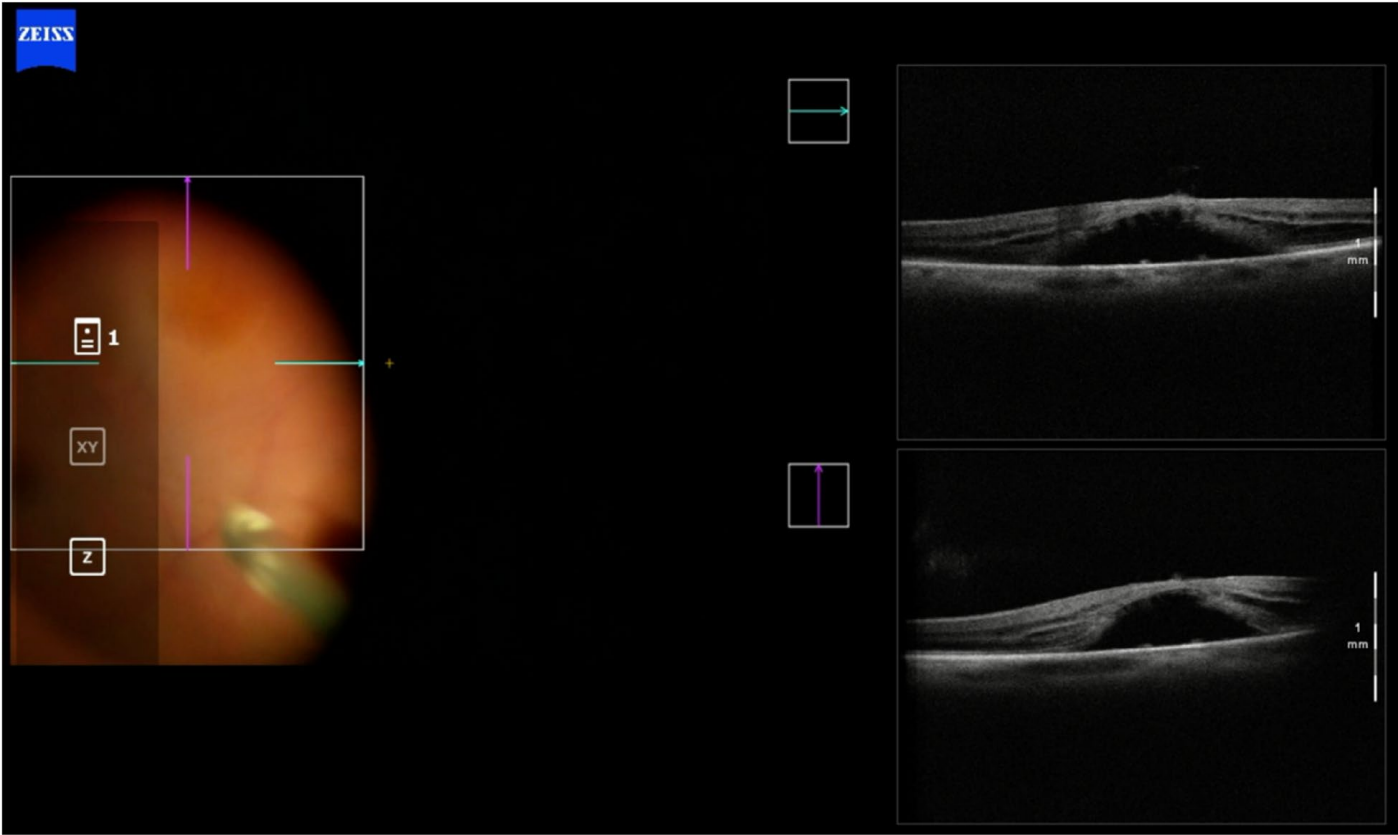
Case 1. IOCT showing the closure of the macular hole without performing the ILM peeling

**Fig. 5:**
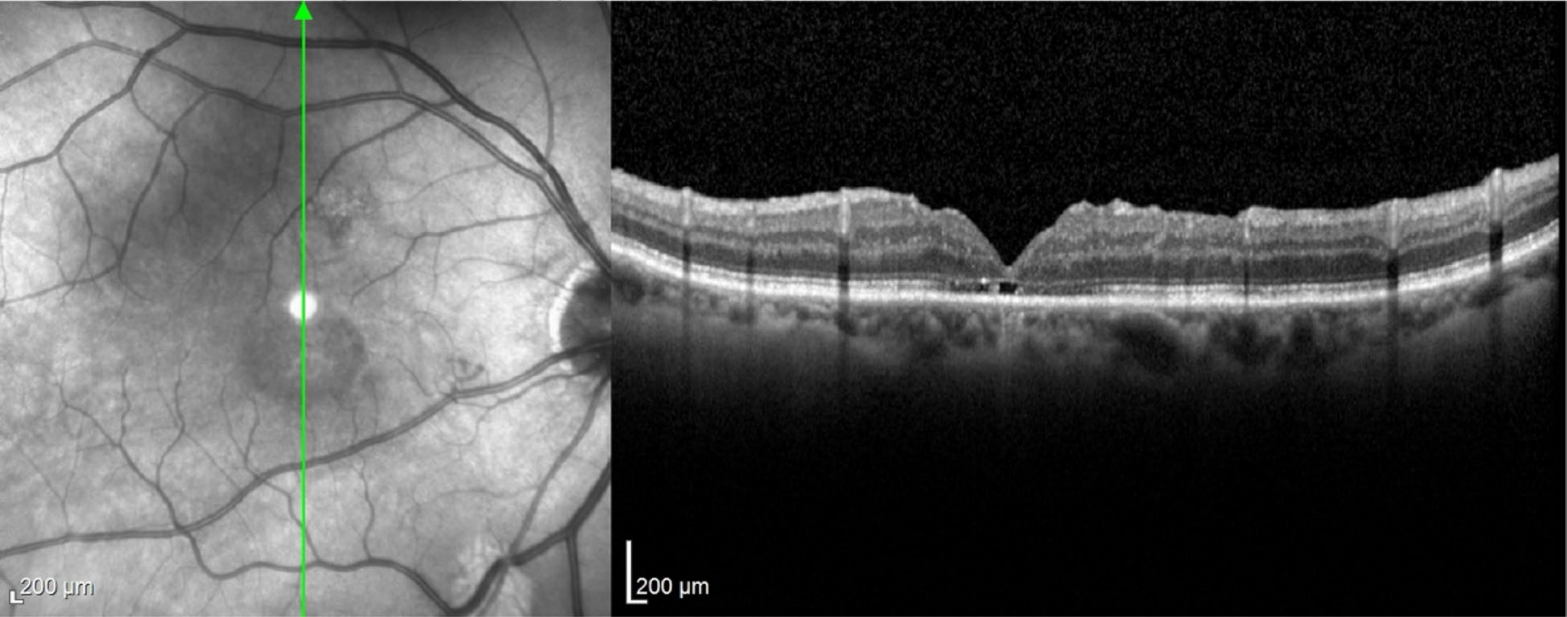
Case 1. Macular hole with 6 months of postoperative closure after posterior vitrectomy associated with the release of vitreomacular traction

In case 1 (Figs 3, 4 and 5), iOCT allowed the avoidance of dye use and potential retinal toxicity, as well as the unnecessary ILM peeling, thus preventing unnecessary surgical trauma. In this case, iOCT enabled a change in technique and intraoperative management, as it directly showed the closure of the macular hole after relieving the antero-posterior traction, with postoperative follow-up showing macular hole closure and a visual acuity of 20/200.

**Fig. 6:**
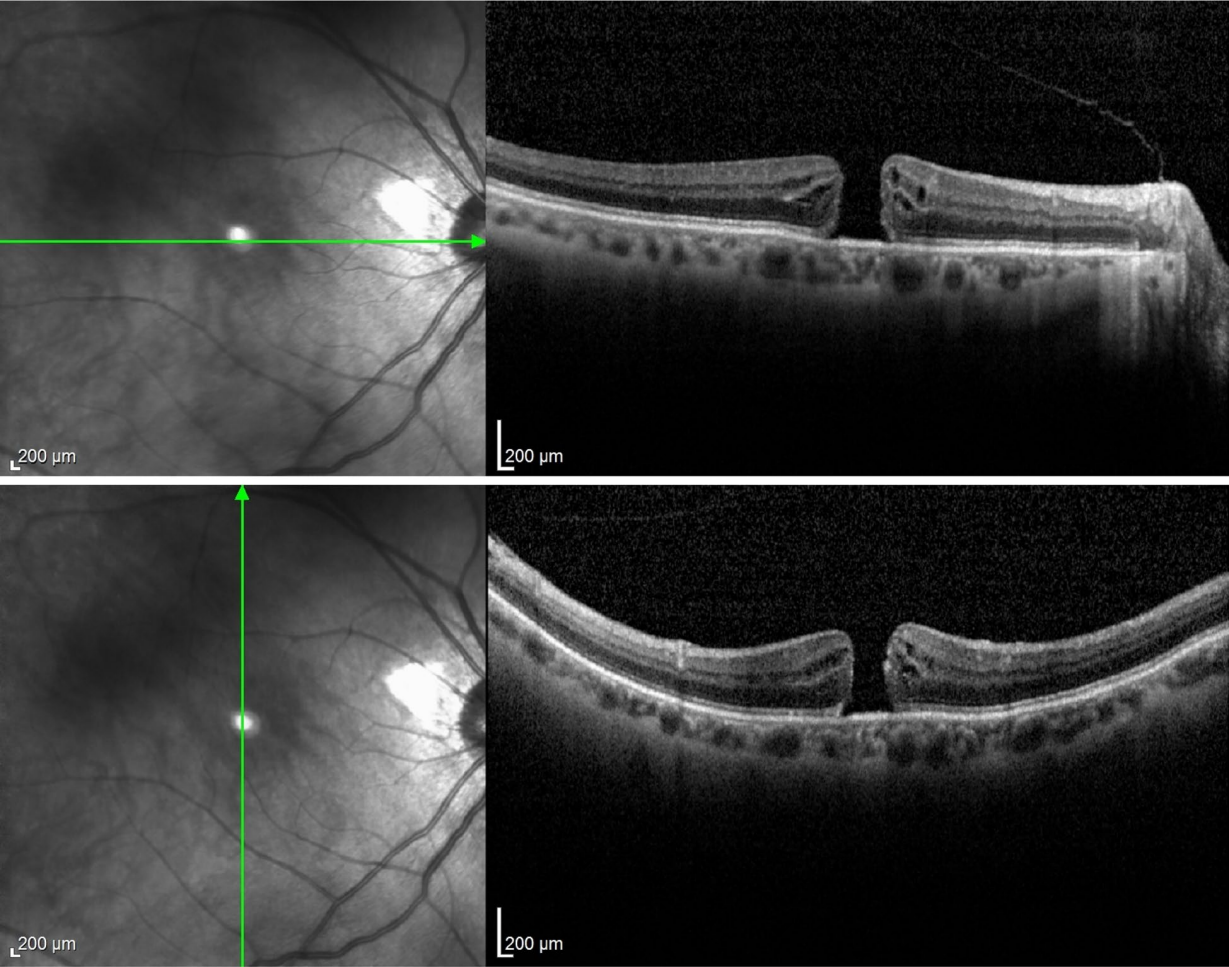
Case 2. Pre-operative OCT showing macular hole

**Fig. 7:**
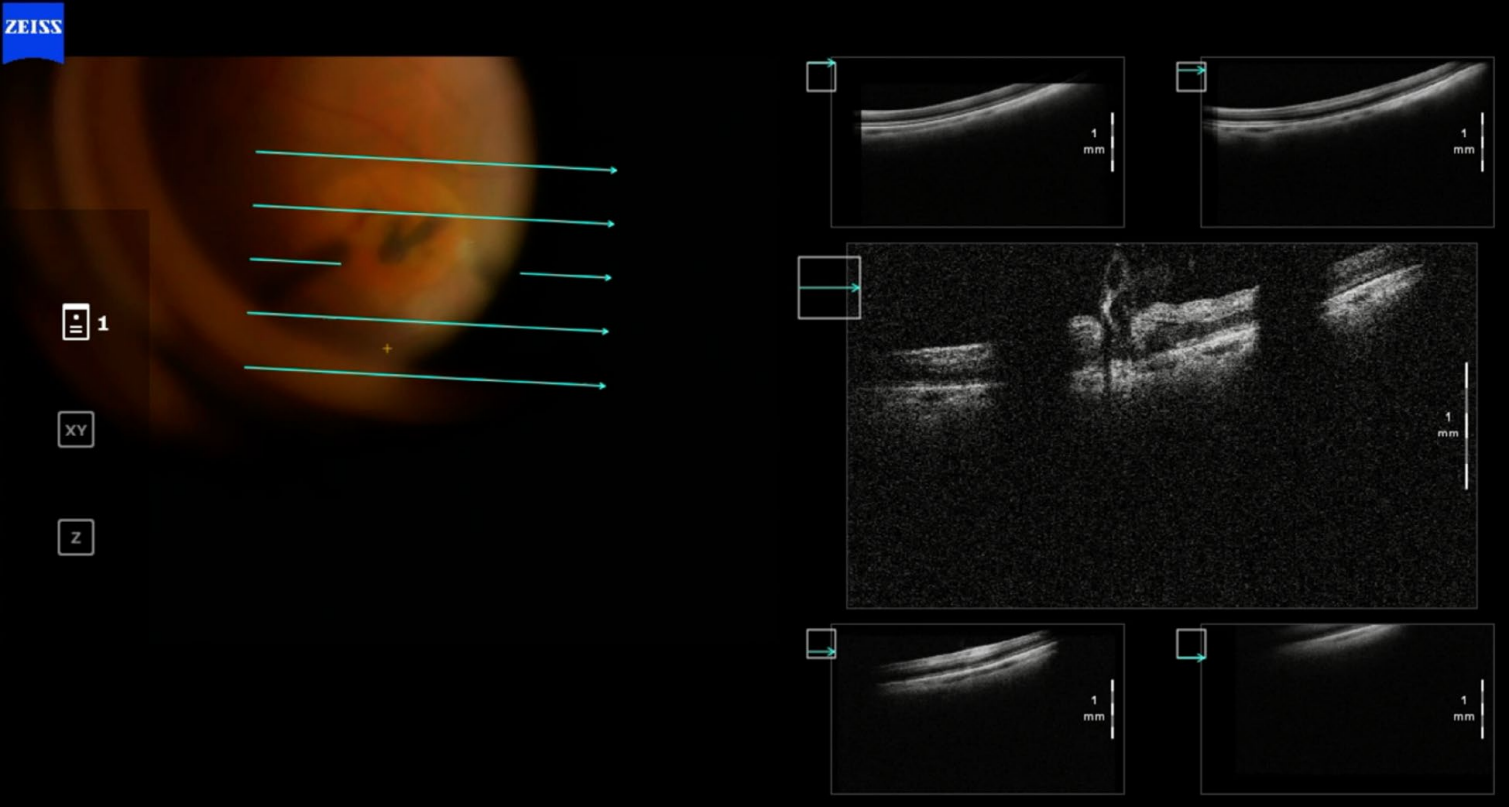
Case 2. IOCT guiding and confirming the correct positioning of the free flap inside the macular hole

**Fig. 8:**
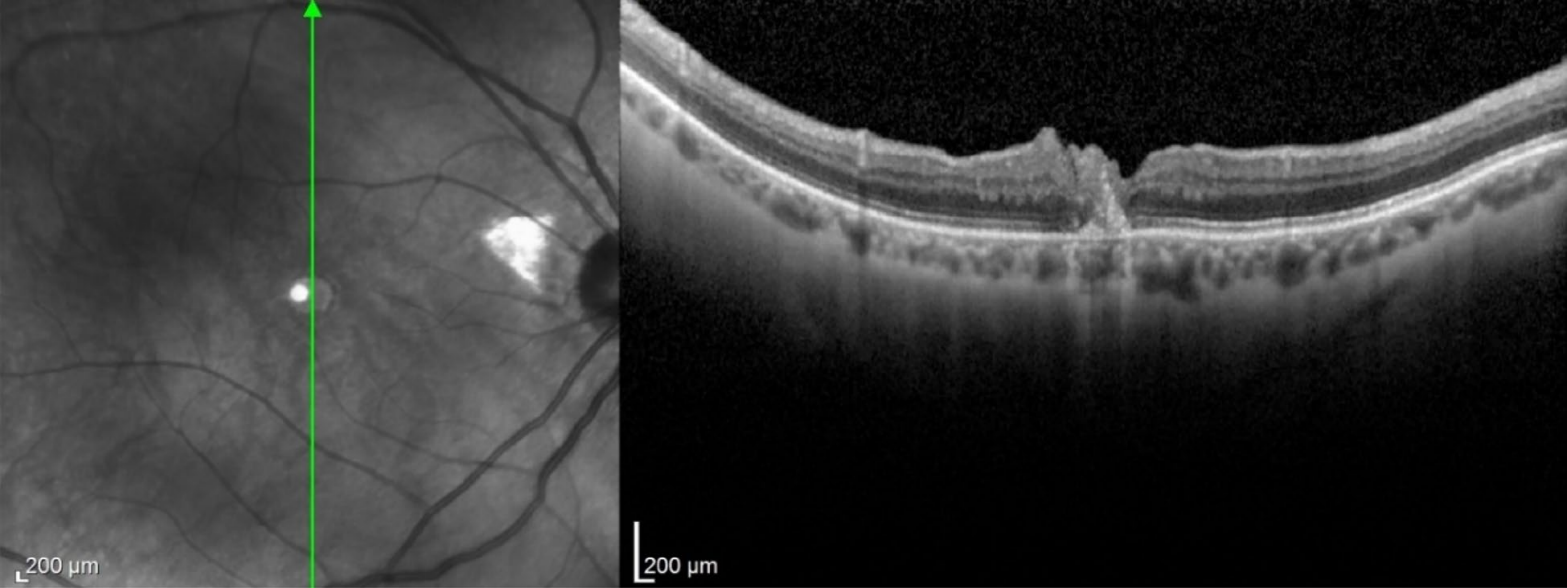
Case 2. Postoperative OCT 6 months after surgery showing closure of the macular hole with the free flap integrated into the retinal layers

Case 2 (figs. 6, 7, 8) involved the need for a change in surgical technique during surgery, which was possible due to the assistance and direct visualization provided by i-OCT. The patient initially had a surgical plan for posterior vitrectomy with ILM peeling and a pedicled flap over the hole. During surgery, the plan was changed to a free flap as the pedicled flap insertion at the macular hole edge ruptured. In this case, iOCT was crucial for positioning the free flap at the base of the macular hole at one end and immediately anchoring it at the other end, a maneuver considered challenging and risky without iOCT yet performed without injury to the retinal pigment epithelium (RPE) and choriocapillaris. The enhancement of this surgical time, essentially carried out with the help of iOCT, could bring benefits in cases where the ILM needs to be maintained without a pedicle on the margins of the macular hole. In this case, the technology not only facilitated positioning the free flap but also identified the amputation of a previously prepared flap. After a complete fluid-gas exchange, the correct flap positioning on the macular hole surface was observed using iOCT. It is interesting to note the ability to observe this positioning even in the presence of air or gas in the posterior cavity. It is speculated that a thin lamina of BSS on the vitreomacular surface enables its visualization through iOCT. This finding is reported here for the first time and warrants further exploration in similar situations. At the end, a mixture of 13% C3F8 gas was used, and the patient progressed with complete macular hole closure with the free flap integrated into the retinal layers (Fig. 13).

**Fig. 9:**
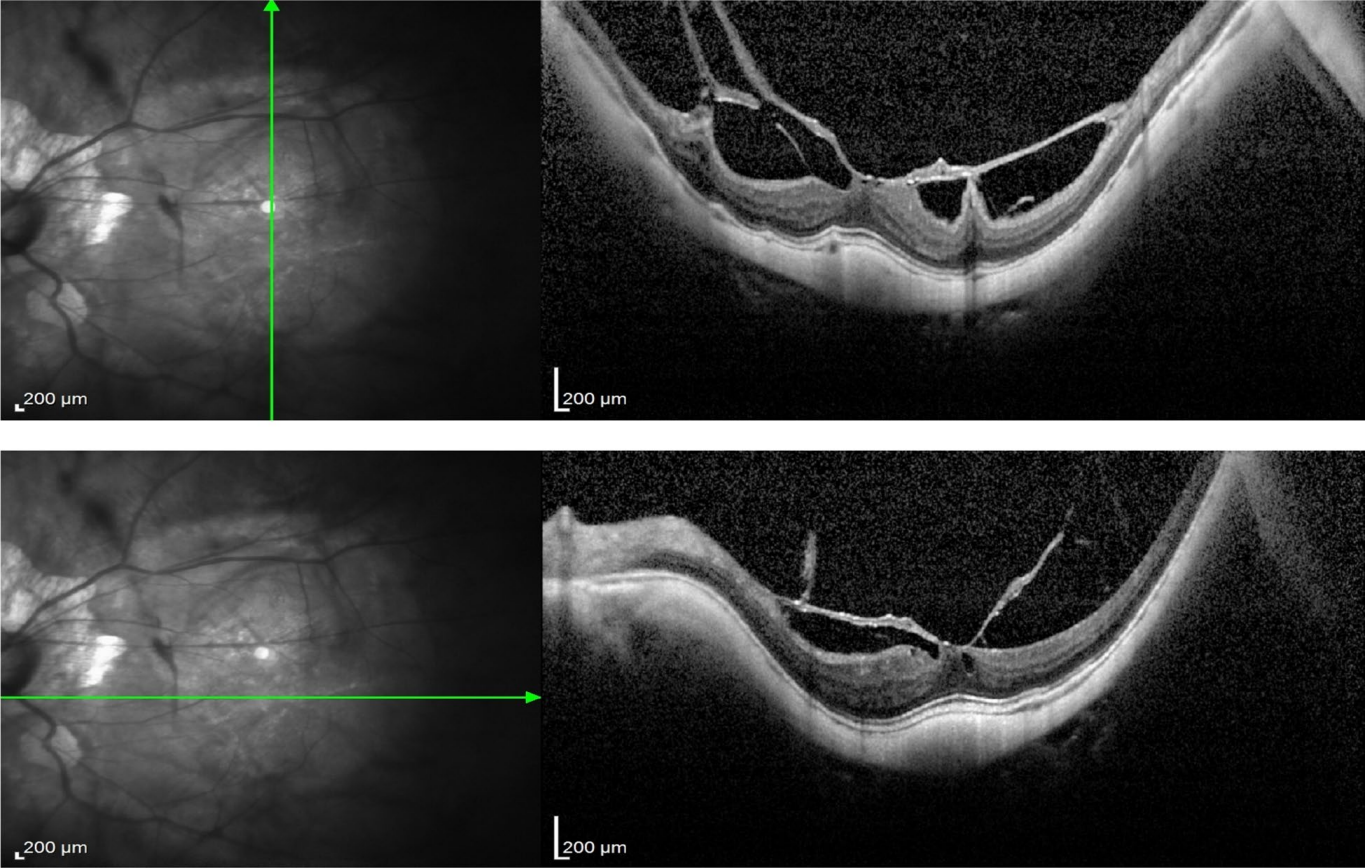
Case 3. OCT of the macula showing severe myopic maculoschisis

**Fig. 10:**
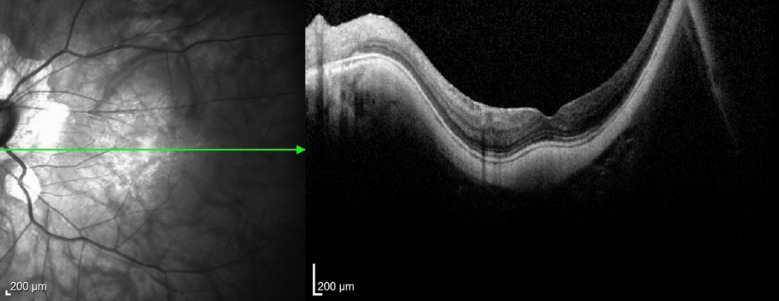
Case 3. OCT of the macula 5 months after the first vitrectomy performed to release vitreoretinal traction due to myopic maculoschisis

**Fig. 11:**
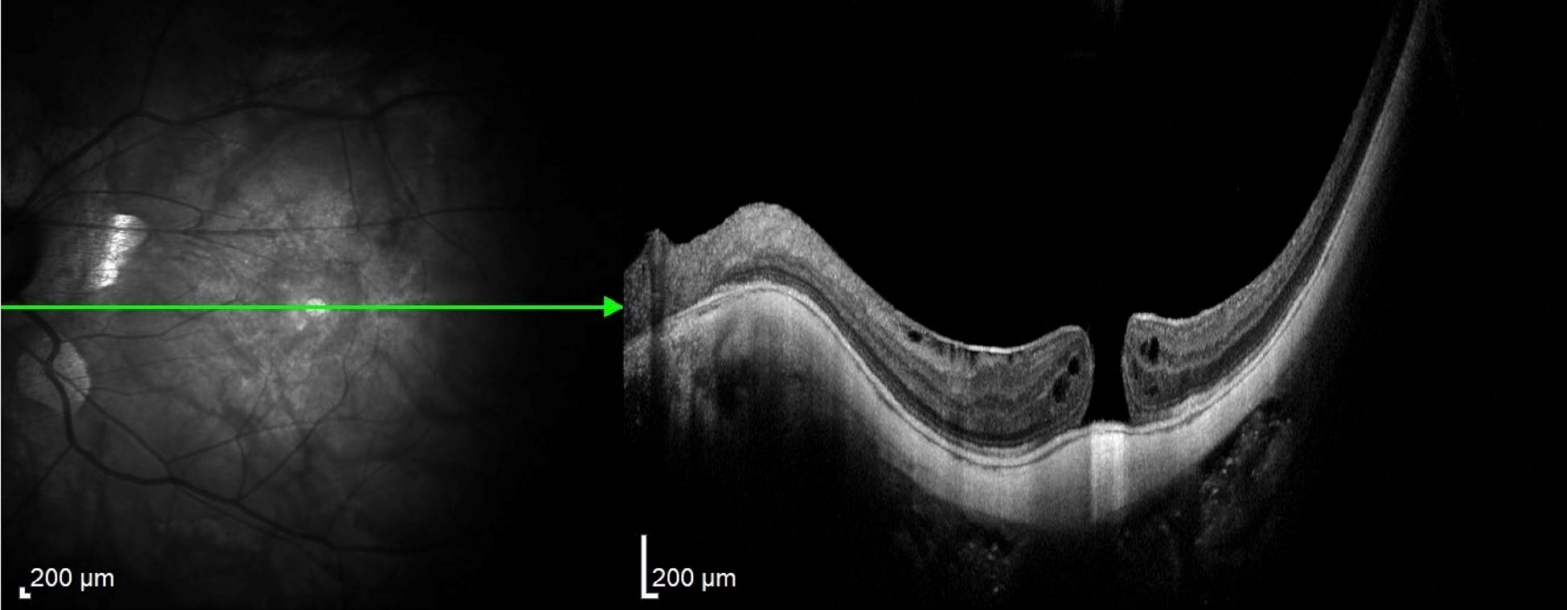
Case 3. OCT showing epiretinal membrane with traction on the inner retinal layers, intraretinal cysts, and full-thickness macular hole in the area of myopic macular staphyloma

**Fig. 12:**
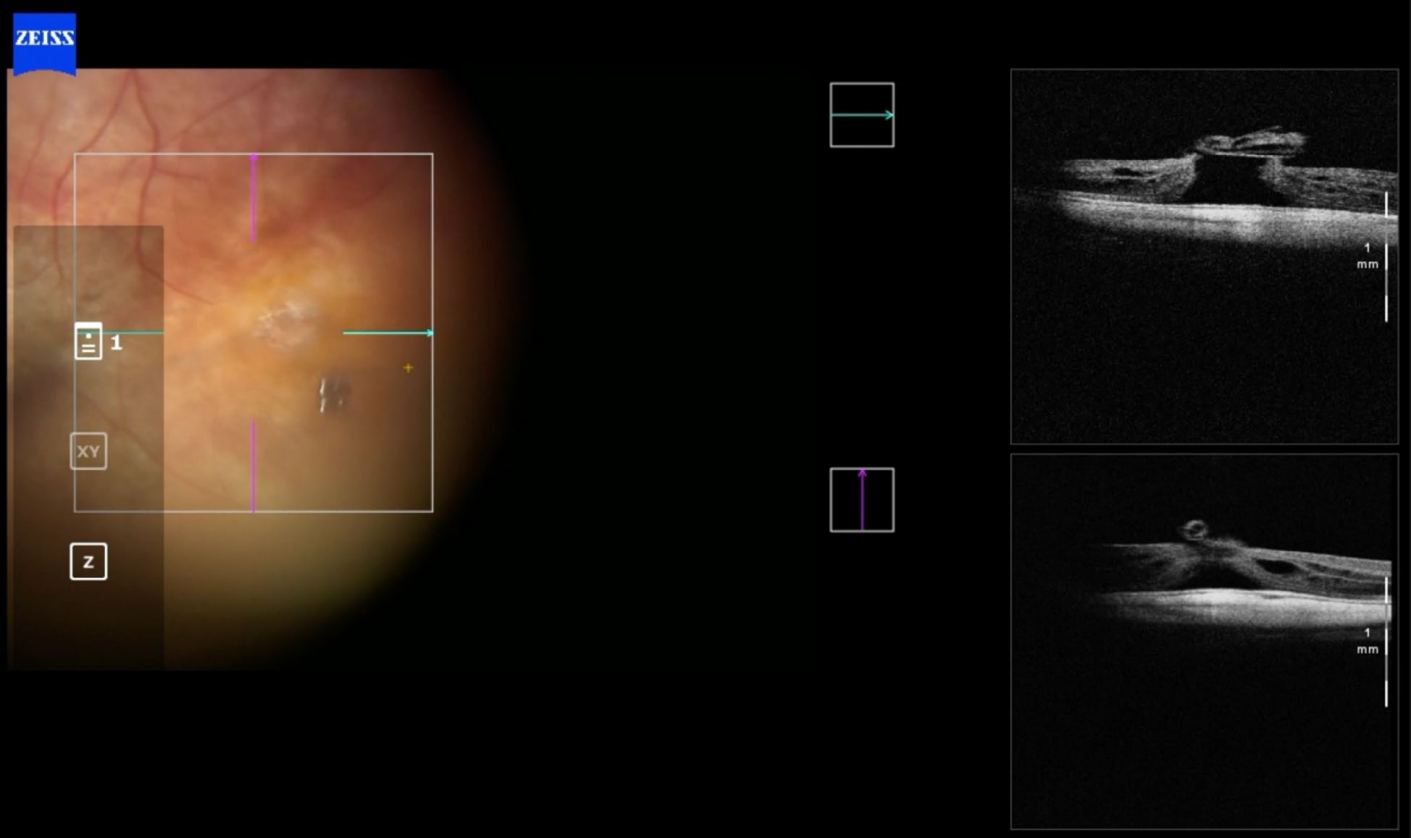
Case 3. IOCT showing a single-layer pedicled flap over the macular hole, anchored in the desired location

**Fig. 13:**
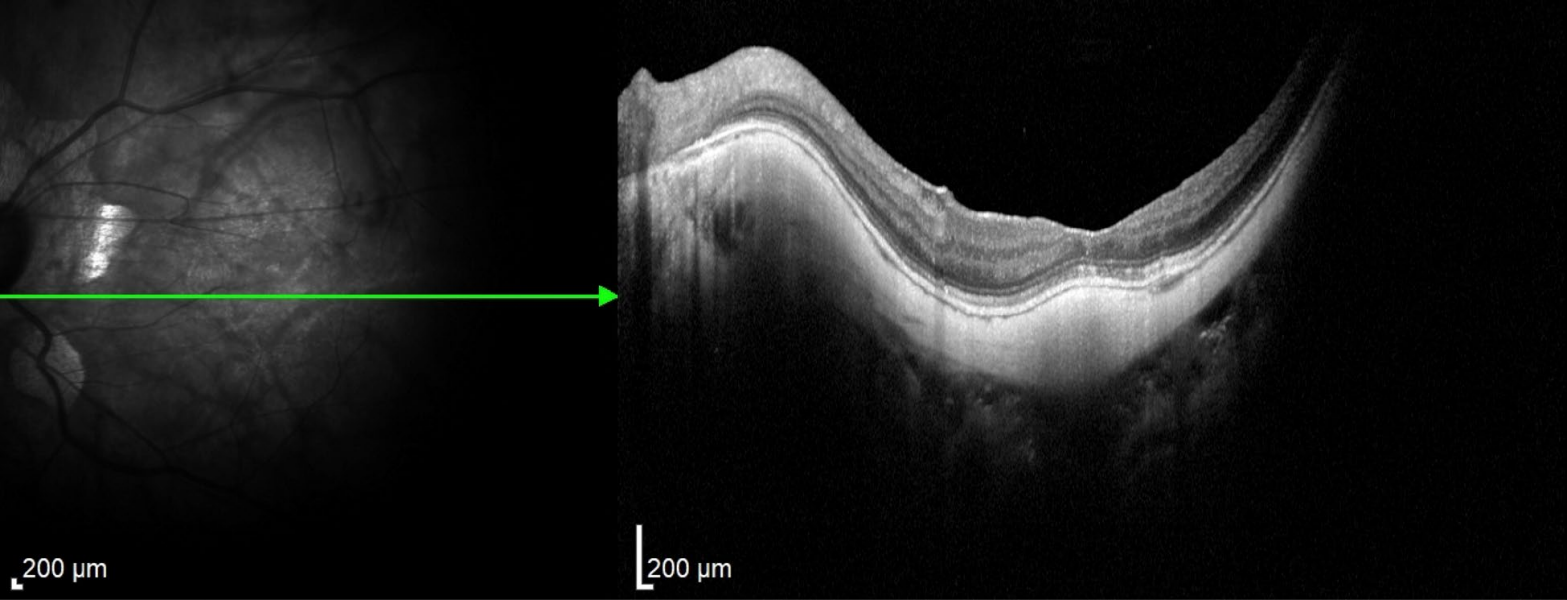
Case 3. Postoperative OCT showing complete closure of the macular hole

**Fig. 14:**
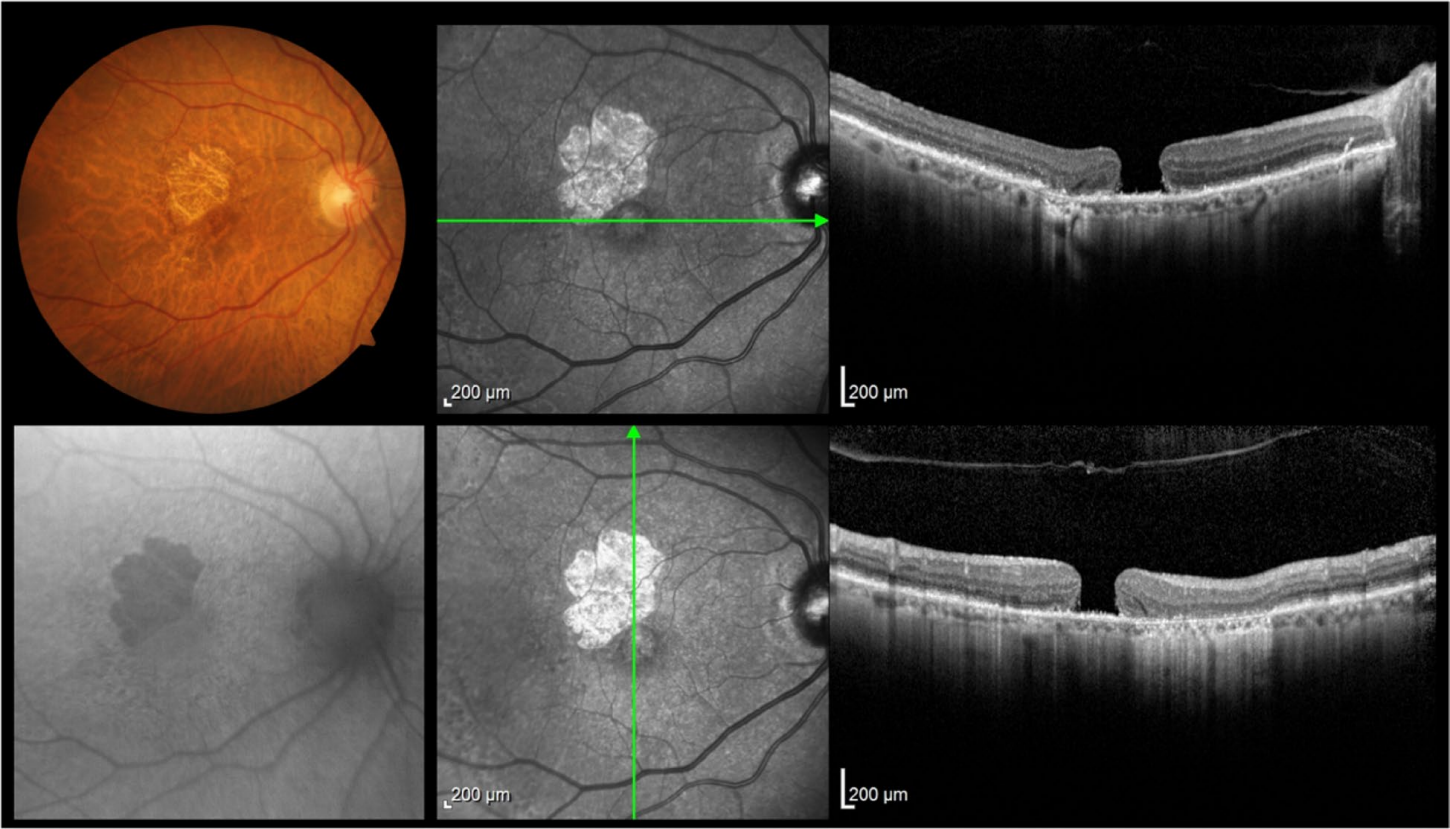
Case 4. Retinography, autofluorescence, infrared, and OCT showing geographic atrophy associated with full-thickness macular hole

**Fig. 15:**
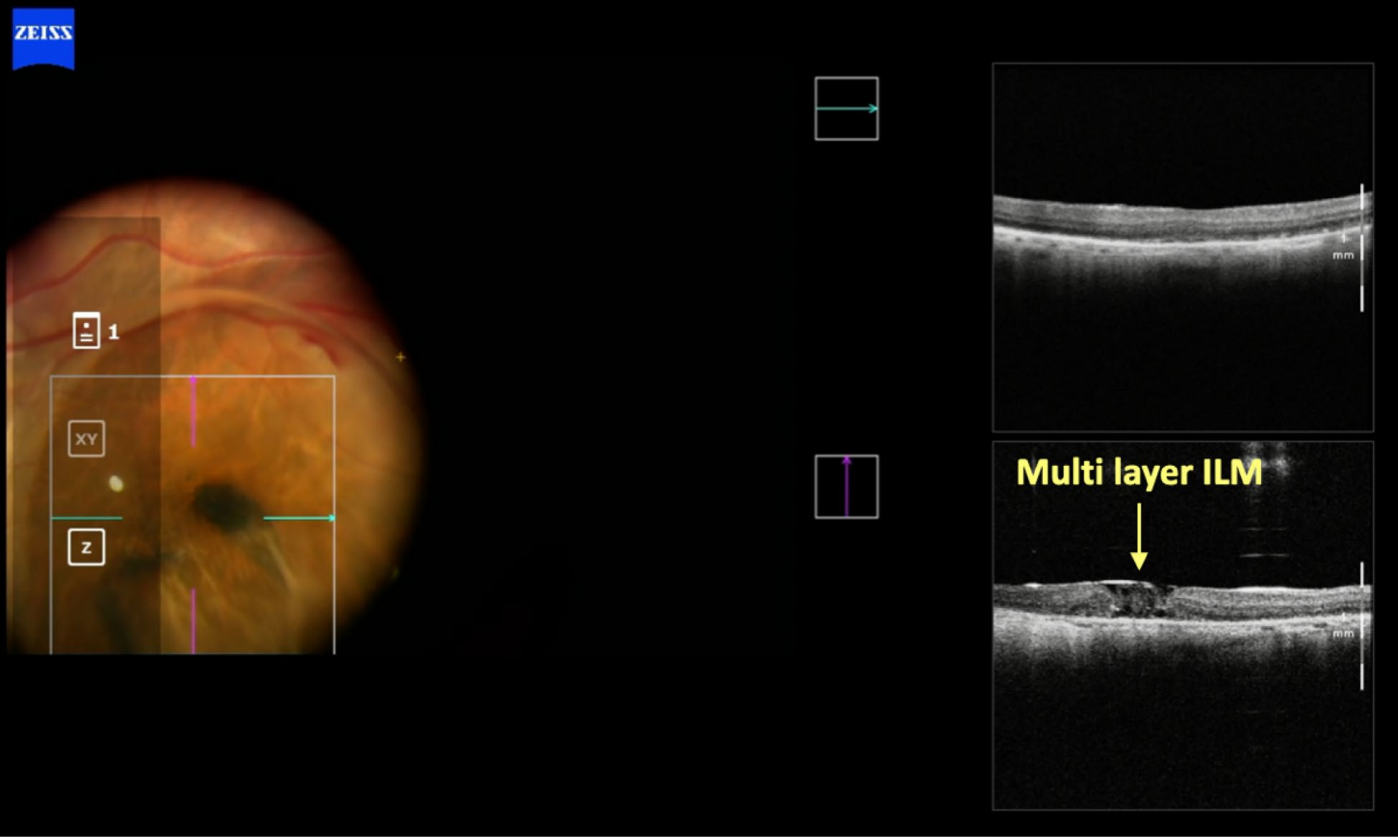
Case 4. IOCT showing the positioning of the ILM in multiple layers over the macular hole

**Fig. 16:**
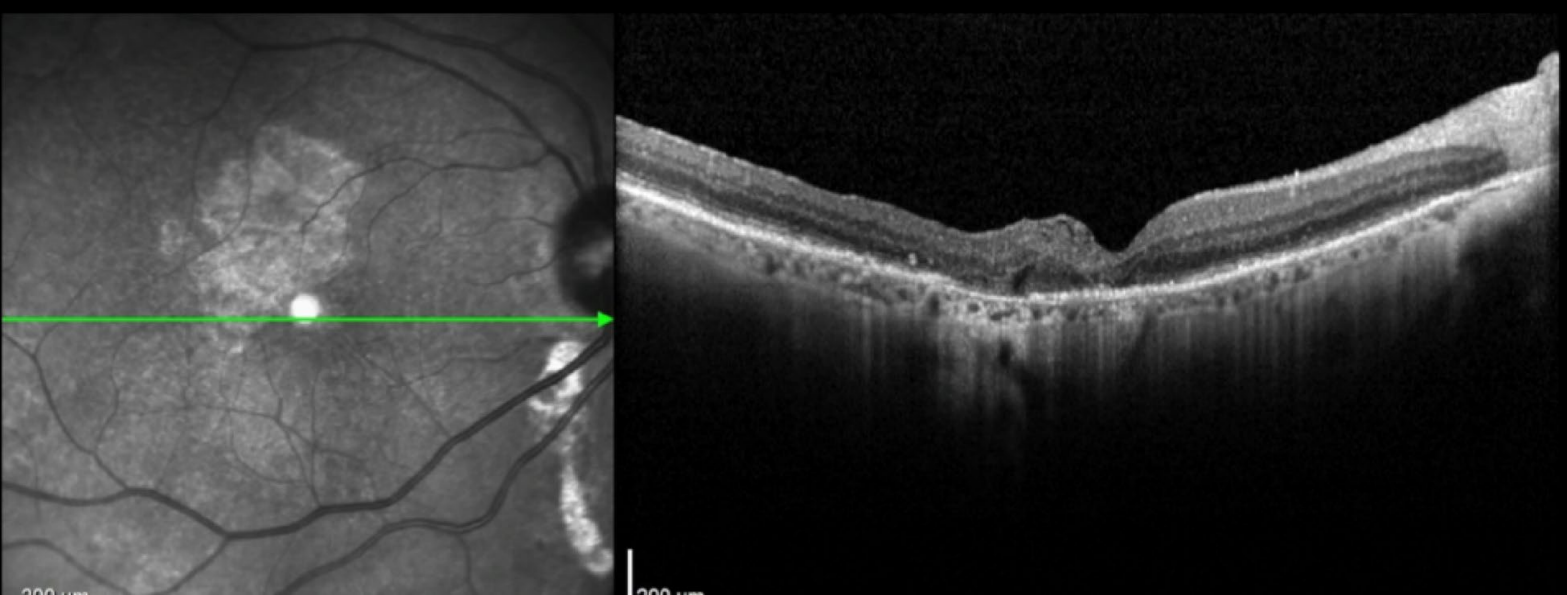
Case 4. Postoperative OCT showing closed macular hole

The removal of ILM in highly myopic patients with retinoschisis/maculoschisis has a considerable rate of iatrogenic ruptures or even macular hole enlargement. Many consider not removing the ILM in these cases, but instead, only separating it around the macular hole. iOCT monitored by the assistant allows assessment of the traction exerted on the macula by the surgeon's maneuvers, which can be minimized with the proper observation reported by the assistant to the primary surgeon, as occurred in case 3 (Figs. 9, 10, 11, 12, and 13). Cases 3 and 4 describe cases treated with ILM peeling and free flap positioning over the macular hole. Previous studies have shown that ILM flaps formed by multiple layers can result in poorer visual outcomes due to excessive gliosis [19]. There was favorable anatomical outcome with complete macular hole closure; however, in case 4 (Figs. 14, 15, 16), the functional result was worse than expected, probably due to the underlying disease (dry AMD with geographic atrophy), which causes irregularities in the outer retinal layers, limiting visual recovery. The relationship between macular hole closure, visual recovery, and the number of ILM layers composing the flap, as described in cases 3 and 4, may be associated with higher or lower macular hole closure rates, as well as higher or lower visual recovery rates. Future investigations will be able to define these anatomical and functional variations using iOCT by analyzing a larger number of cases and extended periods.

**Fig. 17:**
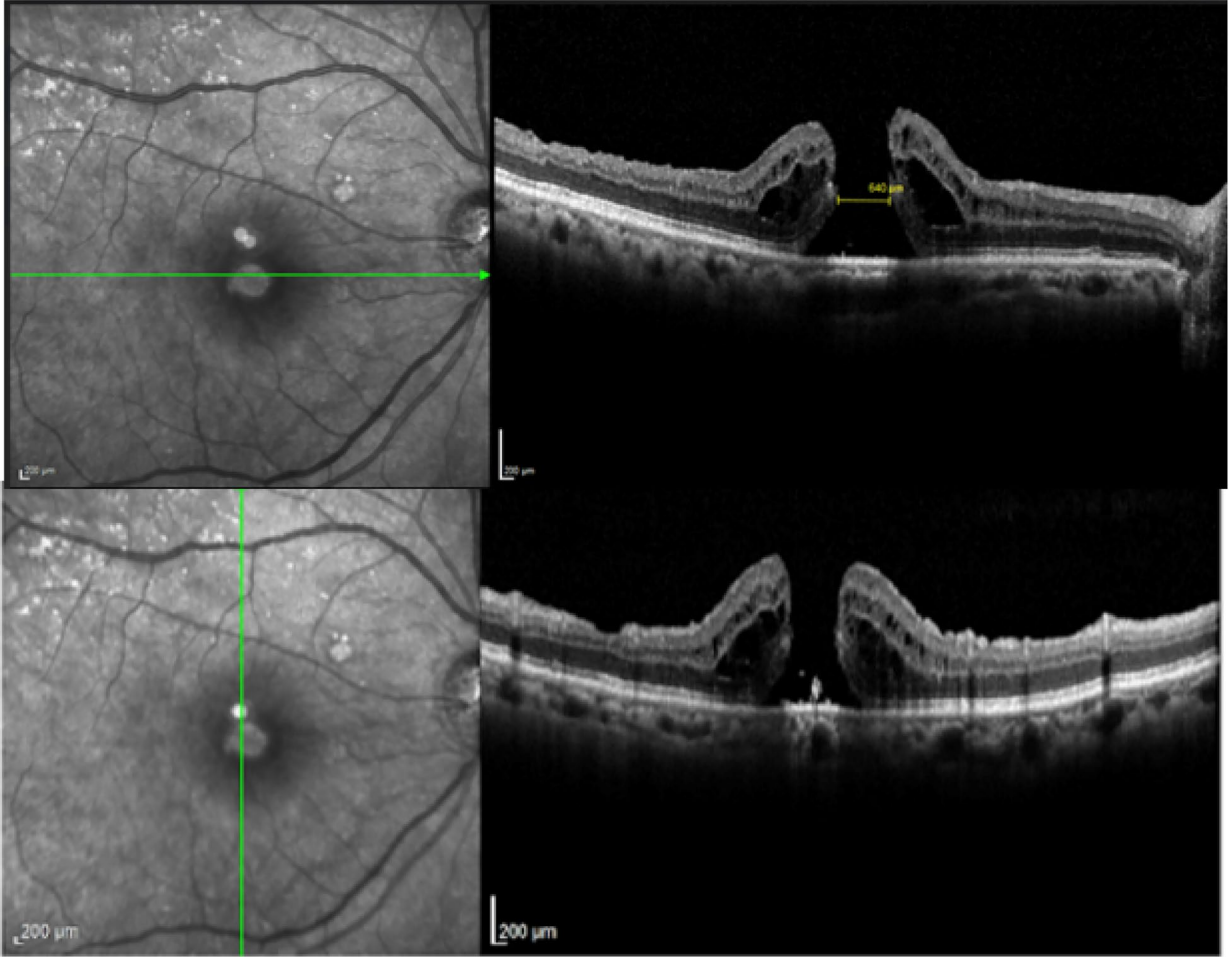
Case 5. OCT showing a full-thickness macular hole with intraretinal cysts in the foveal area. The infrared image beside the OCT shows the margins of the previously performed ILM peeling in the macular region

**Fig. 18:**
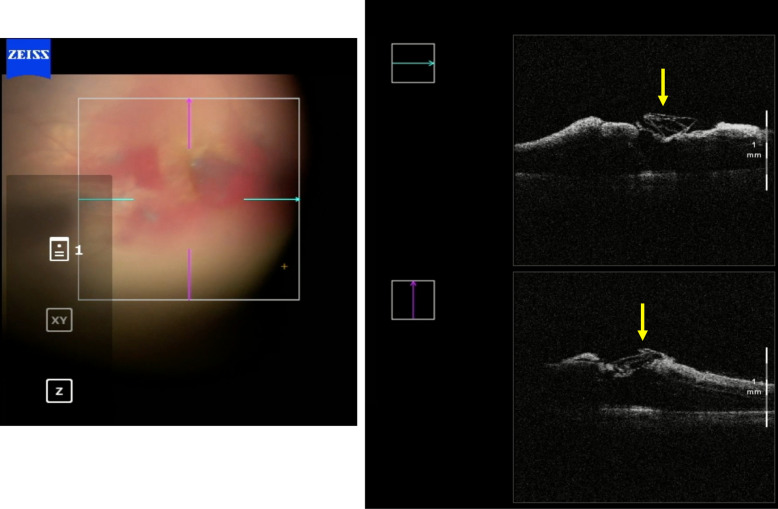
Case 5. iOCT images showing the anchoring of the free flap inside the macular hole by the blood clot. The correct positioning was confirmed using iOCT (yellow arrow)

**Fig. 19:**
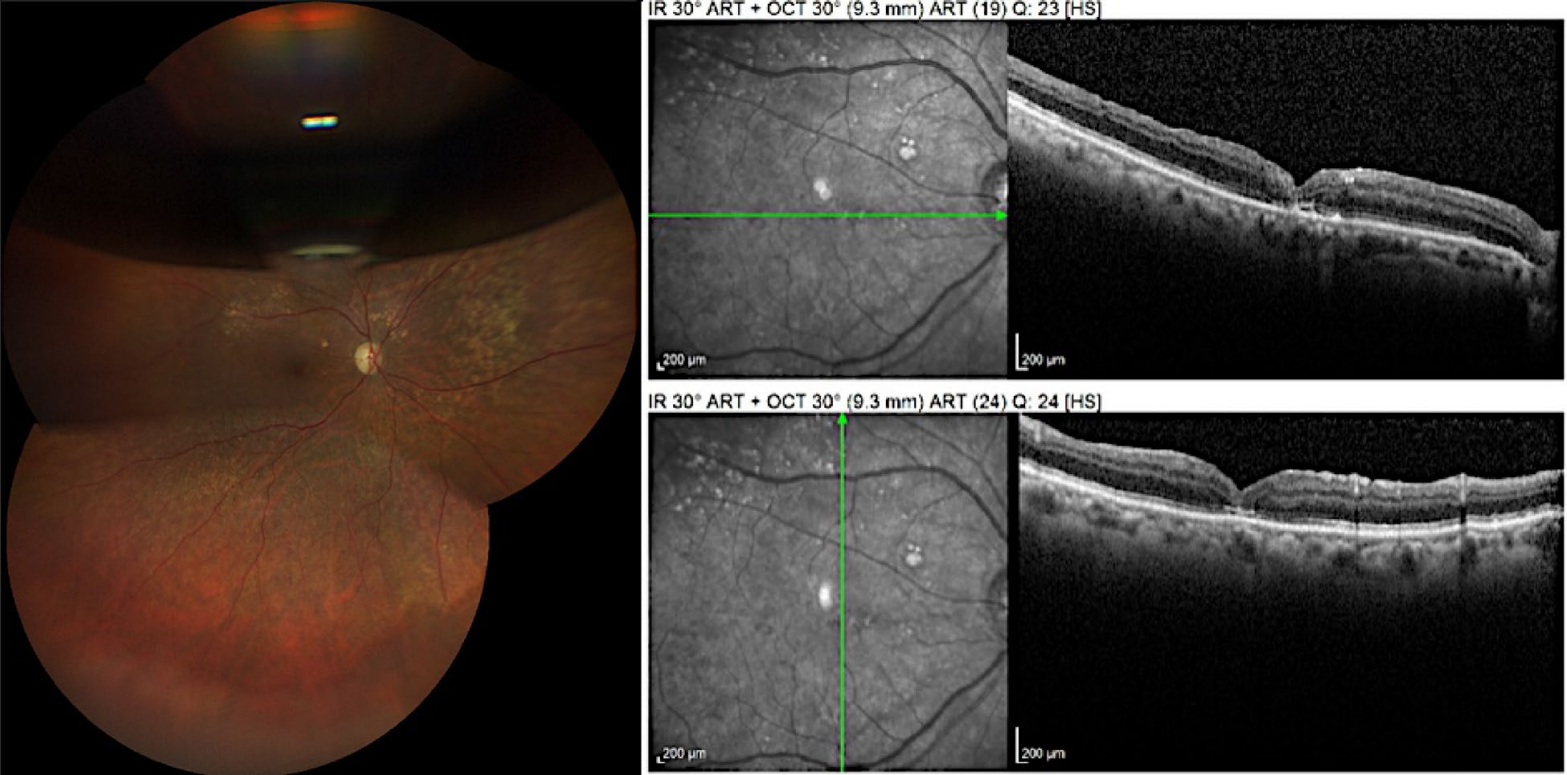
Case 5. Retinography and OCT showing complete closure of the macular hole

**Fig. 20:**
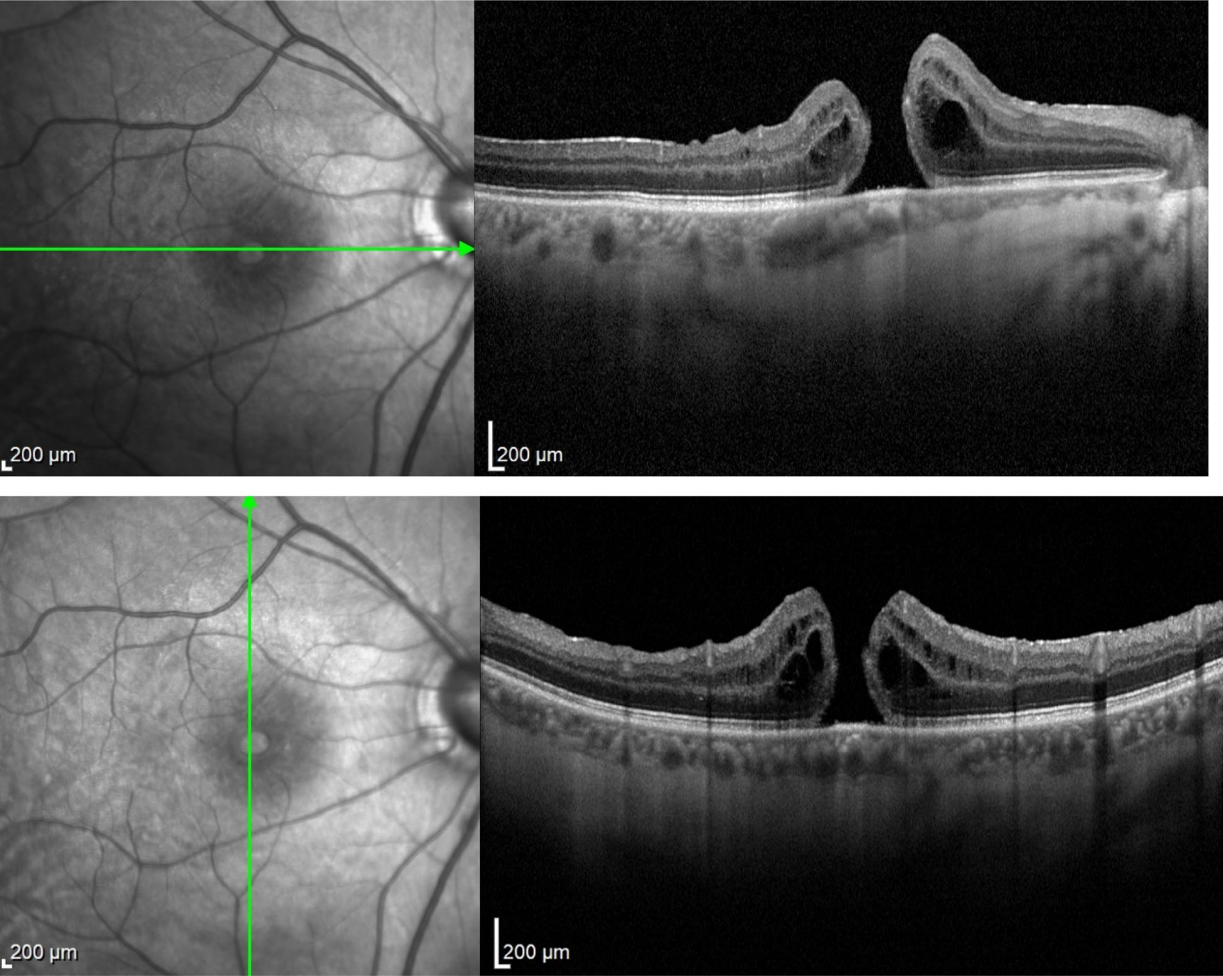
Case 6. Pre-operative OCT showing macular hole

**Fig. 21:**
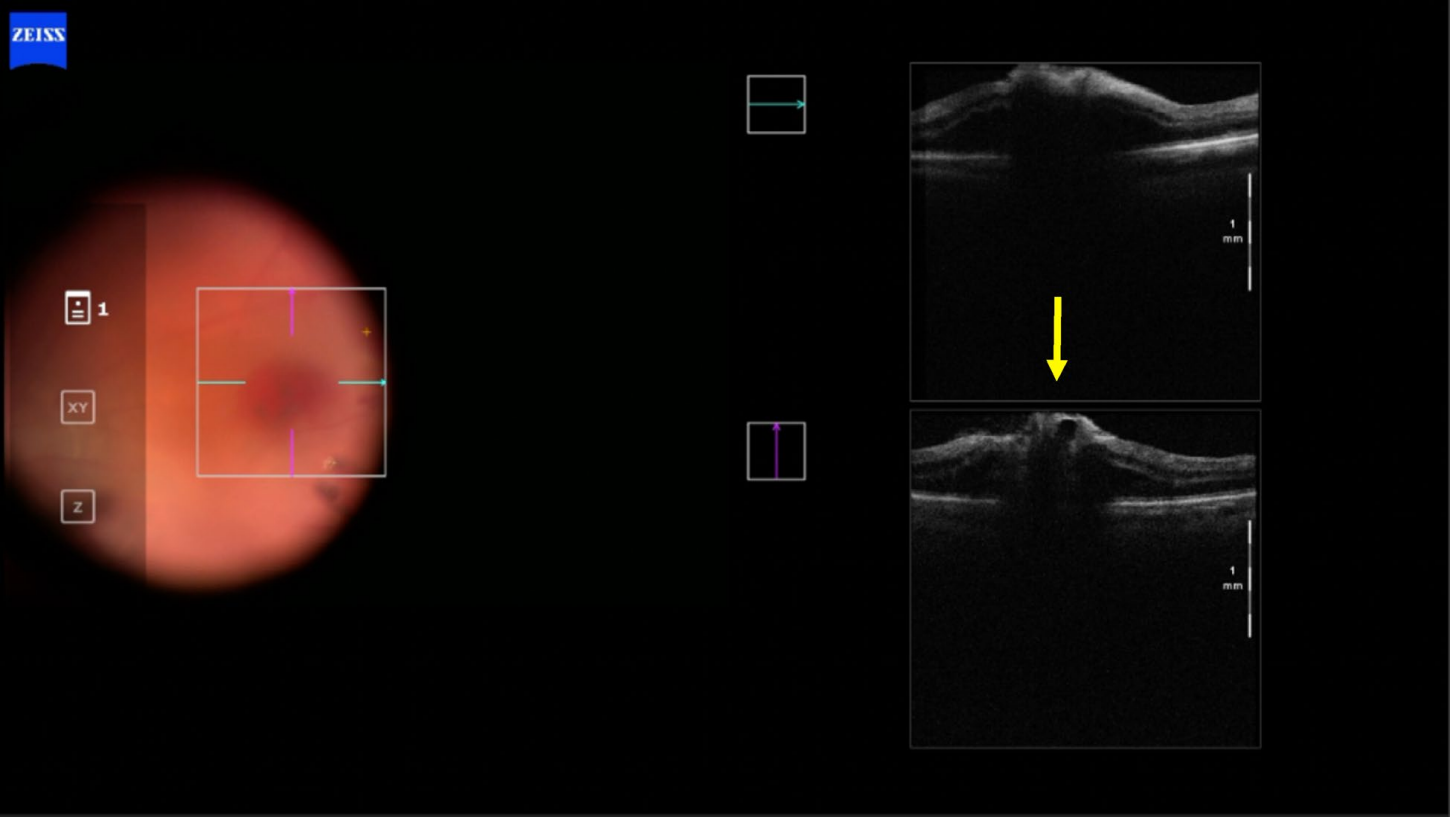
Case 6. iOCT showing ILM over the macular hole immersed in a blood clot (yellow arrow)

**Fig. 22:**
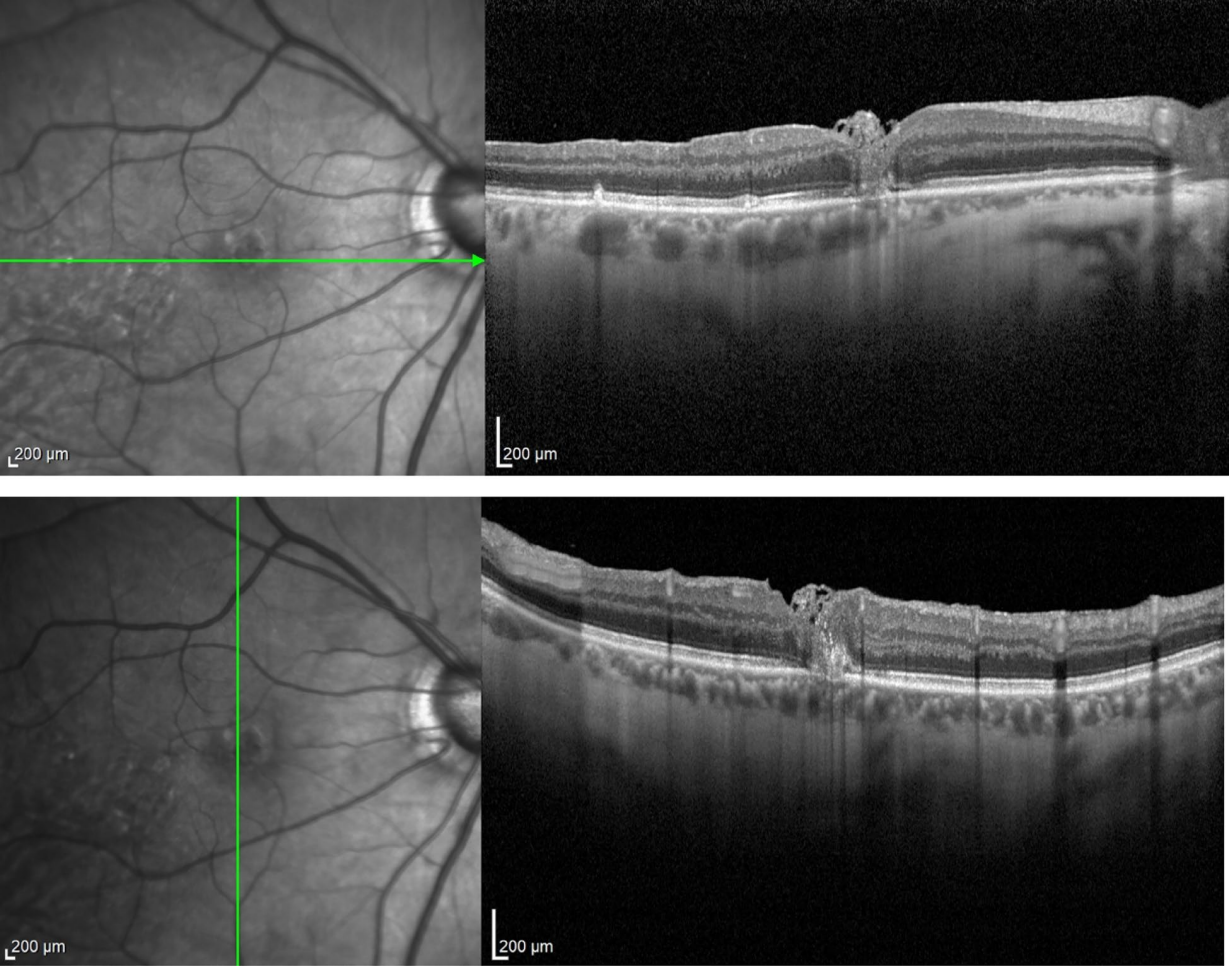
Case 6. Postoperative OCT showing closure of the macular hole with ILM positioned inside the macular hole

Cases 5 (Figs. 17, 18, 19) and 6 (Figs. 20, 21, 22) describe a technique, presented by our group, that enables the use of autologous blood obtained directly from the retinal circulation, facilitating the maintenance of the ILM free flap in the desired position and inducing macular hole closure. A study published in 2015 [11] in which ILM was placed at the macular hole base and a drop of autologous blood was placed on the surface did not yield good results because the cavity was still filled with balanced saline solution, increasing the risk of flap loss. Additionally, placing the ILM first, and not the blood drop, reduces gliosis necessary for flap integration into the retinal layers. The technique presented here was successfully used in three cases of this study. An additional advantage of this technique is that, instead of using blood from the antecubital vein, a small drop of blood from the retinal circulation is used via low-flow micro vessels, facilitating execution and reducing contamination risk. Moreover, the blood drop is placed on the macular hole before the ILM flap is positioned, reducing the risk of displacement once anchored by the clot. IOCT was crucial for identifying the flap integrated into the small drop of blood in the desired position.

Out of the 25 eyes analyzed, 14 were phakic (56%) and 3 (12%) underwent phacoemulsification surgery with intraocular lens implantation. Therefore, 40% of patients still had cataracts at the time of the last follow-up, which may have hindered the proper assessment of the best achievable visual acuity.

The macular hole closure rate with the use of a standard microscope and traditional surgery (posterior pars plana vitrectomy with the use of dyes and associated with internal limiting membrane peeling) ranges from 85 to 100% for idiopathic macular holes [20, 21]. In line with this, the macular hole closure rate in the present study was 92%, with no intraoperative adverse events related to the use of technology, endorsing the feasibility of using iOCT and its additional benefits in macular hole surgery. In our study, 8% of patients did not achieve macular hole closure after the first surgery, a figure comparable to the literature, which reports a small percentage of macular hole persistence after conventional surgery [20]. It is important to mention that this series of cases included recurrent macular holes after previous vitrectomy and myopic maculoschisis, which carry a worse surgical prognosis.

In this study, tamponade was performed with silicone oil in 8% of patients and perfluoro propane gas in 92% of cases. Silicone oil was chosen in two cases. The first case was a patient with a recurrent macular hole, extensive fibrosis, and subretinal membranes, for which a free flap was placed over the macular hole, and silicone oil tamponade was used for a longer-lasting tamponade and an attempt to achieve definitive closure. The second case involved a macular hole associated with retinal detachment, for which silicone oil tamponade was selected due to this associated ocular comorbidity at that time.

Finally, the present study highlighted that the use of iOCT can enhance current surgical practices, significantly impacting intraoperative surgical decisions by providing structural details that could reduce the risk of iatrogenic injuries and personalize patients treatments in cases of macular holes of multivariate etiologies and characteristics.

There were important limitations, such as the lack of randomization with a control group, which only allowed for a specific, targeted analysis of the impact of iOCT usage. Despite this, the study contributes to the literature by endorsing the use of iOCT for posterior segment surgeries, as there were no complications attributed to the visualization system, and no case was converted to the use of a traditional microscope during the surgeries.

## Conclusions

The use of iOCT integrated into the digital microscope for treating macular holes demonstrated excellent usability and effectiveness in visualizing structures and making intraoperative decisions during posterior pars plana vitrectomy, as noted by the surgeon's questionnaire. It also proved to be safe, with no intraoperative complications or adverse effects associated with the technology. It is difficult to compare our macular hole closure rates with previous reports in the literature due to the multivariate etiology and characteristics, some of which carry a worse surgical prognosis. Despite this, the technology evaluated here resulted in a macular hole closure rate consistent with published results in the current literature using conventional surgical microscopes for primary idiopathic macular holes surgeries.

In 36% (9/25) of cases, iOCT directly influenced the final surgical outcome, highlighting the importance of the experienced retinal and vitreous surgeon in identifying critical points and alerting the primary surgeon to changes in the vitreoretinal interface. The confirmation rate of complete internal limiting membrane peeling was 100% for cases where this maneuver was performed. Responses to the questionnaire from the surgeon, assistant, and fellows demonstrated excellent visualization of the surgical field and image quality, improved ergonomics due to the upright neck position, and enhanced teaching through greater depth of field and enlarged images.

The additional time spent by the surgeon using iOCT averaged 3.24 min per surgery.

## Supplementary Information

Supplementary material 1.

## Data Availability

No datasets were generated or analysed during the current study.

## Abbreviations

iOCT: Intraoperative optical coherence tomography
MH: Macular hole
ILM: Internal limiting membrane
OCT: Optical coherence tomography
RPE: Retinal pigment epithelium
3D: Three dimensions
2D: Two dimensions
HUD: Heads-up display
CBCO: Brazilian eye surgery center
UFG: Federal University of Goiás
TCLE: Informed consent form
ERM: Epiretinal membrane
AMD: Age-related macular distrophy
SD-OCT: Spectral-domain optical coherence tomography
SOM: Standard operating microscope
