# Supplementary material for: Pioneering evaluation in Brazil of microscope-integrated optical coherence tomography with a three-dimensional digital visualization system during pars plana vitrectomy for the treatment of macular hole
Source: Int J Retina Vitreous. 2025 May 19;11:57. doi: 10.1186/s40942-025-00671-8 (PMC12087076; doi:10.1186/s40942-025-00671-8)
Supplement: Supplementary file 1 — Supplementary material 1. [file 40942_2025_671_MOESM1_ESM.docx]

**ANNEX I - Evaluation/Questionnaire for Surgeon, Assistant, and Fellows**

1. What score would you give for the visualization of the surgical field?

2. What score would you give for the image quality?

3. Regarding the usability of the technology and any difficulties encountered: What score would you give?

4. Was there an improvement in ergonomics, lighting, and interaction with the assistant team and fellows in training?

5. In this case, was the presence of a specialist assistant surgeon important to review critical points and issue alerts to the primary surgeon?

6. Did the use of iOCT impact intraoperative decisions (flap creation, extension, etc.)?

7. Were there any intraoperative complications attributed to the use of the technology?

8. **Questionnaire for Fellows:**

- Was your learning experience the same, better, or much better when comparing the analog microscope with 2D screen visualization to 3D surgery?

- Were you able to see the fine maneuver dimensions? YES or NO?

- Were you able to observe the effectiveness of delicate maneuvers? YES or NO?

- Were you able to perceive the 3D images throughout the process? YES or NO?

**Legend:**

Score

0 - Very Poor

1 - Poor

2 - Fair

3 - Good

4 - Very Good

5 - Excellent
